# Reviewer Fatigue in Peer Review: A Need for New Strategies to Overcome

**DOI:** 10.4314/ejhs.v35i5.1

**Published:** 2025-09

**Authors:** Eyob Girma Abera, Daniel Yilma

**Affiliations:** 1 Department of Public Health, Jimma University, Jimma, Ethiopia; 2 Department of Internal Medicine, Jimma University, Jimma, Ethiopia

Peer review is a fundamental element of scientific publishing, upholding standards of rigor, transparency, and credibility prior to new knowledge being included in the scholarly record. Nonetheless, the system is experiencing significant strain due to what has been widely identified by editors as “reviewer fatigue” (1). Across various disciplines, there has been an increase in reviewers declining invitations, refusal, submitting feedback that is shorter or less comprehensive, and delaying the return of their reports. According to a 2023 study, reviewer fatigue is a growing issue that negatively affects both the quality and speed of reviews (2). This trend poses risks to both the quality and timeliness of scientific publication, highlighting the need for effective and sustainable solutions to support the peer review process.

Reviewer fatigue stems from multiple causes. A major factor is the global increase in manuscript submissions, which has led to a greater demand for reviewers. Moreover, peer review is mostly voluntary and offers little formal recognition or reward. It is rarely counted toward academic promotion or funding evaluation, and it is often deprioritized in favor of more career-rewarding tasks. Academics already manage busy schedules filled with teaching, research, and administrative responsibilities, so reviewing papers adds to their unpaid workload. Additionally, a small group of highly active reviewers often handle much of the burden, while newer or less prominent scholars are rarely asked to participate, resulting in an unequal distribution of reviewing tasks within the research community. Structural challenges in the publication process add to reviewer fatigue. Many journals draw from the same group of experts, who often receive several review requests at once from different publishers (2). Additionally, poorly prepared manuscripts and unethical practices by some authors like submitting the same work to multiple journals can complicate matters. When submission systems are confusing or inefficient, it also increases frustration for everyone involved.

Reviewer fatigue impacts the entire publication process. Editors find it harder to get reviews done quickly, which leads to delays in making decisions and sharing research. This causes authors to feel frustrated and uncertain, and journals may face lower quality control if reviewers rush or provide shallow feedback. Ultimately, these issues can erode trust in the peer review system—an essential part of scholarly communication.

To reduce reviewer fatigue, journals can broaden the reviewer pool to include early-career researchers and graduate students under supervision. Co-reviewing offers training while distributing workload. Editors should monitor invitations and avoid overburdening individuals. Streamlining via clear guidelines, standardized checklists, triage systems, and sharing reviews through “portable peer review” reduces redundancy (3). Recognising reviewers with certificates, acknowledgments, or incentives like fee discounts also boosts engagement. AI tools can also support editors by matching reviewers, detecting plagiarism, and checking methods, increasing efficiency without replacing human oversight (4).

Solving reviewer fatigue requires both new procedures and shifts in academic culture. Peer review must be acknowledged by institutions, funders, and journals as valuable scholarly work worthy of recognition. Authors who benefit from peer review should also contribute to it, ensuring fair responsibility. A sustainable system depends on cooperation within the research community and a collective commitment to uphold peer review integrity.
